# Chlorine in Combination with Phenic Acid—The Ideal Antiseptic and Disinfectant

**Published:** 1892-08

**Authors:** 


### Selections and Abstracts.

### CHLORINE IN COMBINATION WITH PHENIC ACID —THE IDEAL ANTISEPTIC AND DISINFECTANT.

J. E. Chambers, M. D., coroner's physician of St. Louis, speaks as follows on this subject:

The term "antiseptic" is no longer doubted as a medical term; nor is there longer a doubt of the value of antiseptics in surgery, midwifery, or the hygiene of the sick room.

The victory of antiseptics is the greatest triumph achieved by scientific medicine in an age of triumphs, and so numerous are the antiseptic preparations that have sprung up, that the question of what antisepsis is, changes to the question of the selection of an antiseptic agent.

The nearer we keep to nature, the fewer mistakes we make. In calling nature to our aid, we find that chlorine in the shape of the various chlorides, is nature's great antiseptic. Were it not for the chloride of sodium—common salt—in any ocean, it would be one seething mass of corruption, and the present invigorating sea-breeze would create a pestilence capable of removing all the higher orders of life from the face of the earth.

Ages before the terms "septic" or "antiseptic," were known or used, our ancestors were practicing antisepsis with chloride of sodium, without knowing the philosophy of its action, and were nearer to nature than many scientists of our scientific era. The antiseptic property of chloride of sodium is inherent in the chlorine; sodium, like lime and mercury, having no antiseptic properties. The more ready a chloride is to decompose and give up its chlorine, the more marked are the antiseptic properties of that chloride. All the chlorides are now classified as antiseptics, while the bases of the various chlorides are usually inert, antagonistic to antiseptic action, or so irritating to the tissues as to unfit them for use in medical or surgical practice.

Chlorine has no rival as an antiseptic, but in its free gaseous form it is difficult to manage, while in its compounds are found the objectionable features of the bases, with which it is combined.

Following nature, custom and common sense, it has been a maxim from Biblical days, that "Cleanliness is next to Godliness." Water being easily impregnated with chlorine, "chlorinated water" was highly recommended for cleansing foul ulcers, before anything about antiseptics was known.

The cleansing property of water is well known, and the master of antiseptics for medical and surgical practice is a carbolized chloro-oxide of hydroxyl, known as chloro-phenique. In this preparation are all the elements that go to make up an ideal antiseptic, viz.: carbolic acid in sufficient quantity to anæsthetize the exposed nerve fibres, and to reduce the local temperature, and chlorine gas held in suspension sufficient to destroy all septic material. A ten to twenty per cent. solution is a valuable wash to all ulcerated mucous surfaces, such as the mouth, nose, or throat, or as an injection into vagina, uterus, bladder or rectum, in an ulcerated or inflammatory condition of these organs. Its value as a disinfectant cannot be estimated, as it gives off by evaporation sufficient of its gases to disinfect the sick room, and rid it of all offensive or poisonous odors. If the chlorine odor is offensive to the patient, that objection may be met by any suitable perfume.

Chloro-phenique is strong enough in its undiluted state for any case or condition in surgery or medicine calling for antiseptics or disinfectants, while minor conditions are met by solutions of various strengths.

In summing up the qualities of chloro-phenique we find, first, that in its use we are but following nature's laws; second, that it can be made of any desired strength or solution; third, that its disinfectant are equal to its antiseptic properties, and last, but most important, its volatile properties render the atmosphere sterile in the vicinity of the wound to which it is applied, equal to the action of a constant spray.

In conclusion, allow me to say that chloro-phenique, as an antiseptic and disinfectant, has no superior, and we need no other.—*The International Journal of Surgery.*

